# AT7867 Inhibits Human Colorectal Cancer Cells via AKT-Dependent and AKT-Independent Mechanisms

**DOI:** 10.1371/journal.pone.0169585

**Published:** 2017-01-12

**Authors:** Shihu Zhang, Zhengming Deng, Chen Yao, Ping Huang, Yi Zhang, Shibing Cao, Xiangcheng Li

**Affiliations:** 1 Department of General Surgery, First Affiliated Hospital of Nanjing Medical University, Nanjing, China; 2 Department of General Surgery, Affiliated Hospital of Nanjing University of Chinese Medicine, Nanjing, China; 3 Orthopedic Department, Affiliated Hospital of Nanjing University of Chinese Medicine, Nanjing, China; Universite Paris Diderot-Paris7 - Batiment des Grands Moulins, FRANCE

## Abstract

AKT is often hyper-activated in human colorectal cancers (CRC). This current study evaluated the potential anti-CRC activity by AT7867, a novel AKT and p70S6K1 (S6K1) dual inhibitor. We showed that AT7867 inhibited survival and proliferation of established (HT-29, HCT116 and DLD-1 lines) and primary human CRC cells. Meanwhile, it provoked caspase-dependent apoptosis in the CRC cells. Molecularly, AT7867 blocked AKT-S6K1 activation in CRC cells. Restoring AKT-S6K1 activation, via expression of a constitutively-active AKT1 (“ca-AKT1”), only partially attenuated AT7867-induced HT-29 cell death. Further studies demonstrated that AT7867 inhibited sphingosine kinase 1 (SphK1) activity to promote pro-apoptotic ceramide production in HT-29 cells. Such effects by AT7867 were independent of AKT inhibition. AT7867-indued ceramide production and subsequent HT-29 cell apoptosis were attenuated by co-treatment of sphingosine-1-phosphate (S1P), but were potentiated with the glucosylceramide synthase (GCS) inhibitor PDMP. *In vivo*, intraperitoneal injection of AT7867 inhibited HT-29 xenograft tumor growth in nude mice. AKT activation was also inhibited in AT7867-treated HT-29 tumors. Together, the preclinical results suggest that AT7867 inhibits CRC cells via AKT-dependent and -independent mechanisms.

## 1. Introduction

Colorectal cancer (CRC) is a leading cause of cancer-related mortality around the world [1,2,3]. CRC’s incidence has also been rising, especially in Eastern counties [1,2,3]. But its prognosis has not been improved, particularly for those with advanced and/or metastatic CRC [1,2]. Over the past decades, molecule-targeted therapy has become the research focus for CRC treatment [2,4,5,6]. Groups are developingnoveland more efficient anti-CRC agentsthat target different oncoproteins [2,4,5,6].

AKT, or protein kinase B (PKB), is a serine/threonine kinase that lies downstream of phosphatidylinositol 3-kinase (PI3K) [7,8]. Over-expression and/or hyper-activity of AKT and AKT-regulated signalings are often detected in human CRC [7,8]. AKT plays a pivotal role in a number of cellular behaviors, including cell growth, proliferation and metabolism as well as survival and apoptosis-resistance [7,8]. On the other hand, AKT inhibition, silence or loss-of-function mutation could lead to CRC cell death [9,10]. Therefore, different AKT inhibitors are being evaluated in both pre-clinical and clinical CRC studies [11,12,13,14].

Grimshaw *et al*. has recently developed a dual inhibitor of AKT and p70S6K1 (“S6K1”), named AT7867 [15]. This dual inhibitor was shown to block AKT-S6K1 activation and inhibit human tumor cell proliferation [15].Although the effect of AT7867 on human CRC viability was examined by Grimshaw *et al* [15], this effect remains to be fully characterized. Importantly, the mechanisms underlying AT7867-mediated anti-cancer activity are still illusive [15]. We are interested to know whether there are AKT-independent mechanisms also responsible for AT7867-mediated killing of cancer cells. Here, we provided evidences to suggest that sphingosine kinase 1 (SphK1) inhibition and subsequent ceramide production should also participate in AT7867-induced anti-CRC cell activity.

## 2. Materials and Methods

### 2.1. Chemicals and reagents

AT7867 was obtained from Jun-sheng Biotech (Shanghai, China). The caspase-3 inhibitor z-DEVD-fmk, the caspase-9 inhibitor z-LEHD-fmk and the pan caspase inhibitor z-VAD-fmk were obtained from Sigma (Shanghai, China). AKT inhibitors perifosine, MK2206 and AKT inhibitor II were obtained from Selleck (Shanghai, China). C6 ceramide (C6-Cer) was obtained from Avanti (Alabama, US). L-threo-1-phenyl-2-decanoylamino-3-morpholino-1-propanol (PDMP) and sphingosine-1-phosphate (S1P) were also from Sigma. K6PC-5, a SphK1 activator, was provided by Dr. Ji [16]. All the antibodies utilized in this study were from Cell Signaling Tech (Shanghai, China).

### 2.2. Cell culture

Established CRC cells (HT-29, DLD1 and HCT116 lines) were cultured in Dulbecco's modified Eagle's medium (DMEM) with 10% fetal calf serum (FBS), 2 mM L-glutamine, and 100 mg/mL penicillin/streptomycin. All cell culture reagents were obtained from Gibco (Suzhou, China).

### 2.3. Primary culture of patient-derived colon cancer and epithelial cells

Fresh human colon cancer tissues and surrounding epithelial tissues were separately carefully. Tissues samples were then mechanically dissociated, filtered through a 70-μm strainer, and digested as previously reported [10]. Primary cells were then cultured in the described complete medium [10]. Two lines of primary colon cancer cells and one line of primary colon epithelial cells were established. Experiments and the protocols requiring clinical samples were approved by the Ethics Review Board (ERB) of Nanjing Medical University. The written-informed consent was obtained from each participant. A total of two colon cancer patients (Male, 56/66 years old) administrated in the First Affiliated Hospital of Nanjing Medical University (Nanjing, China) were enrolled. All investigations were conducted according to the principles expressed in the Declaration of Helsinki as well as national/international regulations.

### 2.4. MTT assay

Percentage of viable cells was measured by the routine 3-[4,5-dimethylthylthiazol-2-yl]-2,5 diphenyltetrazolium bromide (MTT) assay as described previously [17].

### 2.5. Clonogenicity assay

As described [17], cells (5 × 10^4^ per treatment) were suspended in agar-containing complete medium or plus AT7867 treatment, which were then added on top of a six-well plate. After 8 days, colonies were stained and manfully counted.

### 2.6. BrdU assay of proliferation

Cells with/out the AT7867 treatment were incubated with BrdU (10 μM). Cells were then fixed, and BrdU incorporation was determined by the BrdU ELISA kit (Roche Diagnostics) according to the attached protocol.

### 2.7. Trypan blue assay of cell death

As described [17], after applied treatment, the percentage of “dead” cells was calculated by the number of the trypan blue stained cells divided by the total cell number.

### 2.8. Quantification of apoptosis by ELISA

After applied treatment, the single strand DNA (“ssDNA”) Cell Apoptosis ELISA Kit was applied to detected denatured DNA in ELISA format to reflect cell apoptosis [18].

### 2.9. Annexin V assay

The adherent and floating cells were collected and washed. Cells were then incubated in Annexin V solution (10 μg/mL, Invitrogen, Shanghai, China) for 15 minutes. Immediately prior to reading on a FACS Calibur flow cytometer (BD, Nanjing, China), 10 μg/mL of propidium iodide (Invitrogen) was added to the mix. Annexin V positive cells were gated as apoptotic cells.

### 2.10. TUNEL assay and caspase activity assay

The detailed protocols of TUNEL staining assay and caspase activity assay were described in detail in other studies [17,19].

### 2.11. Western blot assay

After treatment, both floating and adherent cells were collected and washed. Cells were then harvested using the RIPA buffer (Biyuntian, Nanjing, China). Aliquots of 30 μg lysates per sample were separated by SDS-PAGE and transferred to PVDF membranes (Millipore, Nanjing, China). The blots were blocked and incubated with designated primary and secondary antibodies. Targeted protein bands were visualized with ECL reagents and developed with Hyper-film (GE Healthcare, Shanghai, China). Results were quantified via the ImageJ software (NIH).

### 2.12. AKT1 shRNA knockdown

The two lentiviral AKT1 shRNAs (“-a/-b”), with non-overlapping sequences, were designed by Genepharm (Shanghai, China). The AKT1shRNA (10 μL/mL) or the scramble control shRNA (Santa Cruz Biotech, Nanjing, China) was added to cultured cells for 24 hours. Puromycin (5.0 μg/mL) was then included to select stable colonies for 4–6 passages. The AKT1 knockdown in the stable cells was verified by Western blot assay.

### 2.13. Constitutively-activate AKT1 expression

As previously described [20], cells were seeded onto six-well plate with 50% of confluence. The constitutively-activate AKT1 (ca-AKT1-flag, a gift from Dr. Zhou) [20]plasmid or the control vector was transfected to the cultured cells via the Lipofectamine 2000 reagents (Invitrogen). Stable cells were again selected by puromycin (5.0 μg/mL) for 4–6 passages.

### 2.14. SphK1 activity assay and ceramide content assay

Analyzing sphingosine kinase 1 (SphK1) activity and cellular ceramide content were described in detail in our previous study [17].

### 2.15. *In vivo* tumor studies

HT-29 cells were injected subcutaneous (*s*.*c*.) in the right flanks of female nude mice (from the Experimental Animal Center of Nanjing Medical University, Nanjing, China). Animals were randomized into three groups, and treatment was started with vehicle or AT7867 when established tumors were ~100 mm^3^ in average volume. Control mice received vehicle only (10% DMSO, 90% saline) and treatment mice received 10 or 50 mg/kg AT7867 intraperitoneally (*i*.*p*.). Tumor volumes and body weights were monitored every four days. The tumor size was calculated using the formula: V (volume) = 0.5328 × Long × Width × High (mm^3^). For recording mouse body weight, the estimated tumor weight (tumor volumes × 1g/cm^3^) was subtracted from total weight of each mouse. Mice were maintained under the following conditions: 12-hour dark/12-hour light cycle, 24±2°C temperatures, and 50±10% humidity. Mice were observed extremely carefully throughout the experimental period. The clinical signs of mice were recorded daily, and if the criteria of humane endpoints were met, animals were sacrificed. Humane endpoints were considered as rapid weight loss (>15%), abnormal changes in behavior and motion (social and eating behavior), tumor size greater than 2 cm^3^ or skin problems (wounds or signs of inflammation). If animals reached these endpoints, they were euthanized by exsanguination under 2,2,2-tribromoethanol anesthesia (4 mg/10 g body weight, Sigma). All injections in this study were performed via the above anesthesia method [21]. The protocol was approved by the Nanjing Medical University’s Institutional Animal Care and Use Committee (IACUC) and Ethics Review Board (ERB).

### 2.16. Immunohistochemistry (IHC)

IHC staining protocol was reported previously [10]. Briefly, HT-29 tumors were fixed, processed, and embedded in paraffin. The 4 μm tissue sections were blocked before incubation with primary antibody (p-AKT Ser-473 at 1:50) and horseradish peroxidase (HRP)-conjugated secondary antibody (1: 50), which was then subjected to 3,3’-diaminobenzidine color development.

### 2.17. Statistical analysis

Data were normalized to control values of each assay, and were presented as mean ± standard deviation (SD). Statistics was analyzed by one-way ANOVA followed by a Scheffe’s f-test using the SPSS 18.0 software (Chicago, IL). P< 0.05 is considered statistically significant.

## 3. Results

### 3.1. AT7867 inhibits CRC cell survival and proliferation

**Fig 1:**
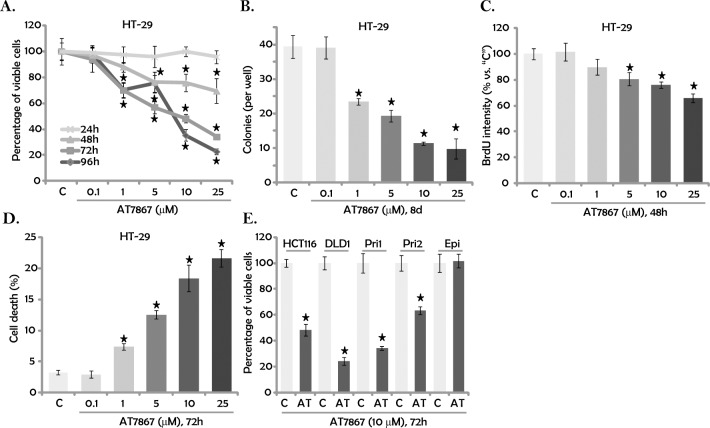
AT7867 inhibits CRC cell survival and proliferation. Established CRC cell lines (HT-29, HCT116 and DLD-1), primary human CRC cells (two lines, “Pri1/2”) and the primary human colon epithelia cells (“Epi”) were treated with/out a range of concentrations of AT7867 for applied time; MTT assay was performed to test viable cell percentage (A and E); Cell proliferation was tested by the clonogenicity assay (B) and the BrdU ELISA assay (C); Cell death was tested by the trypan blue staining assay (D). Data were shown as the mean (n = 5, within the same experiment) with the standard deviation (SD). * P < 0.05 *vs*. “C” (medium control) group.

HT-29 cells were first treated with gradually-increasing concentrations of AT7867 (0.1–25 μM). Cells were then cultured for 24–96 hours, and then cell viability was determined using an MTT assay. Results in Fig 1A illustrated that AT7867, at 1–25 μM, significantly decreased percentage of viable HT-29 cells. AT7867 was dose-dependent in inhibiting HT-29 cell viability (Fig 1A). AT7867 at 0.1 μM was non-cytotoxic (Fig 1A). Notably, it would take at least 48 hours for AT7867 (1–25 μM) to exert significant effect in HT-29 cells (Fig 1A). AT7867-induced viable HT-29 cell decrease could be due to proliferation inhibition and/or simple cell death. Next, clonogenicity assay (Fig 1B) and BrdU incorporation assay (Fig 1C) were performed to test cell proliferation. Results from both assays demonstrated that AT7867 (1–25 μM) significantly inhibited HT-29 cell proliferation (Fig 1B and 1C). For the BrdU incorporation assay, the BrdU ELISA OD results were normalized to MTT OD (Fig 1C). Furthermore, trypan blue assay results showed that AT7867 (1–25 μM) dose-dependently induced HT-29 cell death (Fig 1D).

The potential effect of AT7867 on other human CRC cells was also tested. As demonstrated, AT7867 (10 μM) was cytotoxic when added to the two other established CRC cell lines (HCT116 and DLD-1). It also decreased the percentage of viable patient-derived primary human CRC cells (two lines, “Pri1/2”) (Fig 1E). Remarkably, the AT7867 treatment (10 μM, 72 hours) was non-cytotoxic to the primary colon epithelia cells (“Epi”, Fig 1E). Together, these results demonstrate that AT7867 is cytotoxic and anti-proliferative to cultured human CRC cells.

### 3.2. AT7867 provokes apoptosis in CRC cells

**Fig 2:**
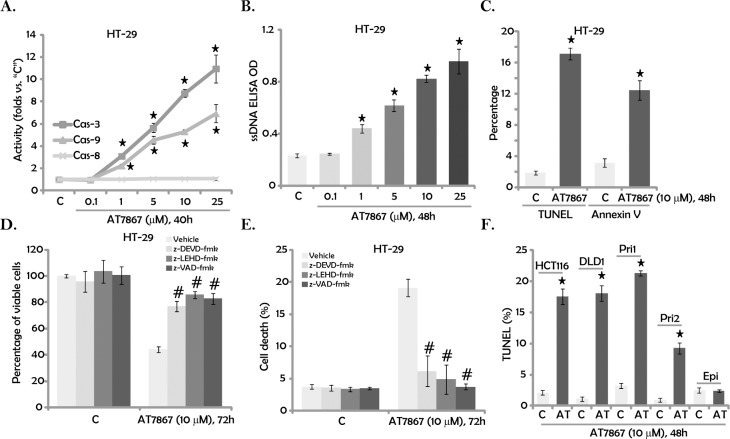
AT7867 provokes apoptosis in CRC cells. CRC cell lines (HT-29, HCT116 and DLD-1), primary human CRC cells (two lines, “Pri1/2”) and the primary human colon epithelia cells (“Epi”) were treated with designated AT7867 (“AT”) for applied time; Activation of caspases (A) and apoptosis (B, C and F) were tested by listed assays; The potential effect of listed caspase inhibitors (40 μM each, 30 min pre-treatment) on AT7867 (10 μM)-induced HT-29 viable cell percentage (D) and cell death (E) was tested. Data were shown as the mean (n = 5, within the same experiment) with SD. * P < 0.05 *vs*. “C” (medium control) group. ^#^ P < 0.05 *vs*. AT7867 only group (D and E).

We next tested the potential effect of AT7867 on CRC cell apoptosis. Under many apoptotic stimuli, cytochrome c released from mitochondria will associate with procaspase-9 and Apaf-1, causing caspase-9 activation [22,23]. The latter further activates caspase-3 and caspase-7 to initiate a caspase cascade, leading to mitochondrial/intrinsic apoptosis pathway activation [22,23]. On the other hand, activation of caspase-8 is the characteristic marker of extrinsic apoptosis pathway activation [22,23,24]. In the current study, we showed that AT7867 (at 1–25 μM) significantly increased the activity of caspase-3 and caspase-9, but not caspase-8, in HT-29 cells (Fig 2A), suggesting activation of intrinsic, but not extrinsic, apoptosis pathway. Furthermore, 1–25 μM of AT7867 also significantly increased the ssDNA ELISA optic density (OD) (Fig 2B). The percentages of TUNEL positive cells and Annexin V positive cells were also increased significantly following AT7867 (10 μM) treatment (Fig 2C). These results demonstrated that AT7867 promoted apoptosis in HT-29 cells.

Next, different caspase inhibitors were applied. z-DEVD-fmk (the caspase-3 inhibitor), z-LEHD-fmk (the caspase-9 inhibitor) and z-VAD-fmk (the pan caspase inhibitor) significantly attenuated AT7867-induced HT-29 cell MTT OD reduction (Fig 2D) and cell death (Fig 2E). These results suggest that AT7867-induced HT-29 cell death requires activation of caspase-3/-9 and apoptosis. Notably, AT7867 (10 μM) was pro-apoptotic to other established (HCT116 and DLD-1) and primary human CRC cells (Fig 2F). Again, no significant apoptosis was detected in AT7867 (10 μM)-treated primary colon epithelial cells (Fig 2F).

### 3.3. AT7867 inhibits AKT-S6K1 activation in HT-29 cells

**Fig 3:**
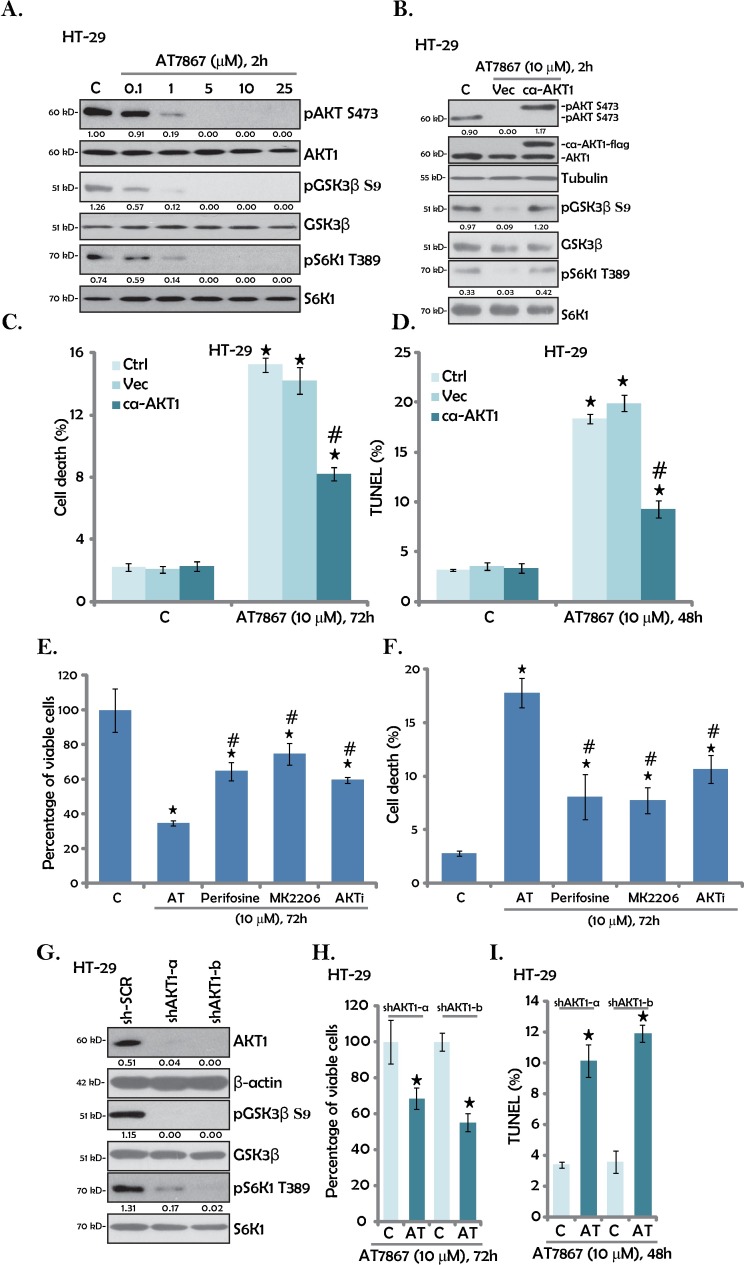
AT7867 inhibits AKT-S6K1 activation in HT-29 cells. HT-29 cells were treated with AT7867 (0.1–25 μM) for two hours. Phosphorylated (“p”) or total AKT1, GSK3β and S6K1 expression was tested by Western blot assay (A). Stably HT-29 cells expressing the constitutively-active AKT1 (“ca-AKT1”) or the empty vector (“Vec”) were treated with/out AT7867 (10 μM) for applied time; Listed kinases expression was tested by Western blot assay (B); Cell death and apoptosis were tested by trypan blue staining assay (C) and TUNEL staining assay (D), respectively. HT-29 cells were treated with 10 μM of AT7867 (“AT”), perifosine, MK2206 or AKT inhibitor II (“AKTi”) for 72 hours, MTT assay was performed to test viable cell percentage (E); Cell death was tested by trypan blue staining assay (F). Stably HT-29 cells with AKT1 shRNA (“shAKT1-a/-b”) or scramble control shRNA (“sh-SCR”) were treated with/out AT7867 (10 μM) for applied time; Expression of listed proteins was tested by Western blot assay (G); Viable cell percentage (MTT assay, H) and apoptosis (TUNEL staining assay, I) were also tested. Kinase phosphorylation (vs. total protein) was quantified (A, B and G). Data were shown as the mean (n = 5, within the same experiment) with SD. * P < 0.05 *vs*. “C” (medium control) group. ^#^ P < 0.05 *vs*. AT7867 treatment of “Vec” group (C and D). ^#^ P < 0.05 *vs*. AT7867 group (E and F).

AT7867 is a newly developed AKT-S6K1 dual inhibitor [15,25]. We thus analyzed AKT-S6K1 signaling in AT7867-treated CRC cells. Results in Fig 3A showed that AT7867 dose-dependently inhibited AKT activation in HT-29 cells. Further, phosphorylated (“p”) GSK3β (S9) and pS6K1 (Thr-389), two major downstream proteins of AKT [8,11], were also largely inhibited following AT7867 (1–25μM) treatment (Fig 3A). Expression of the total proteins (AKT1, GSK3β and S6K1) was not affected by the AT7867 treatment (Fig 3A).

To study the link between AKT inactivation and AT7867-induced actions, a constitutively-active AKT1 (“ca-AKT1”, flag-tagged) [20] was introduced to HT-29 cells. Western blot assay results in Fig 3B confirmedca-AKT1 expression in the HT-29 cells. Remarkably, the ca-AKT1 completely restored AKT activation (pAKT/pGSK3β/pS6K1) in AT7867-treated HT-29 cells, even higher to the control level (Fig 3B, quantification). Yet, it only partially inhibited AT7867-induced HT-29 cell death (Fig 3C) and apoptosis (Fig 3D). Therefore, restoring AKT-S6K1 signaling was not enough to completely rescue HT-29 cells from AT7867, indicating that AKT-independent mechanisms should also play a significant role in mediating AT7867’s cytotoxicity. As a matter of fact, the cytotoxic effect of AT7867 on HT-29 cells was significantly greater than the effects of other known Akt inhibitors, such as perifosine[26], MK2206[27] and AKT inhibitor II (Fig 3E and 3F), although these other AKT inhibitors also blocked AKT-S6K1 activation (Data not shown).

To further support our hypothesis, shRNA strategy was applied to knockdown AKT1 in HT-29 cells. As shown in Fig 3G, the two non-overlapping AKT1 shRNAs (AKT1 shRNA-a/b) both dramatically downregulated AKT1 in HT-29 cells. pGSK3β and pS6K1 were also significantly inhibited (Fig 3G). Interestingly, AT7867 was still cytotoxic (Fig 3H) and pro-apoptotic (Fig 3I) in AKT1-silenced HT-29 cells.

### 3.4. AT7867 inhibits SphK1 to promote ceramide production in CRC cells

**Fig 4:**
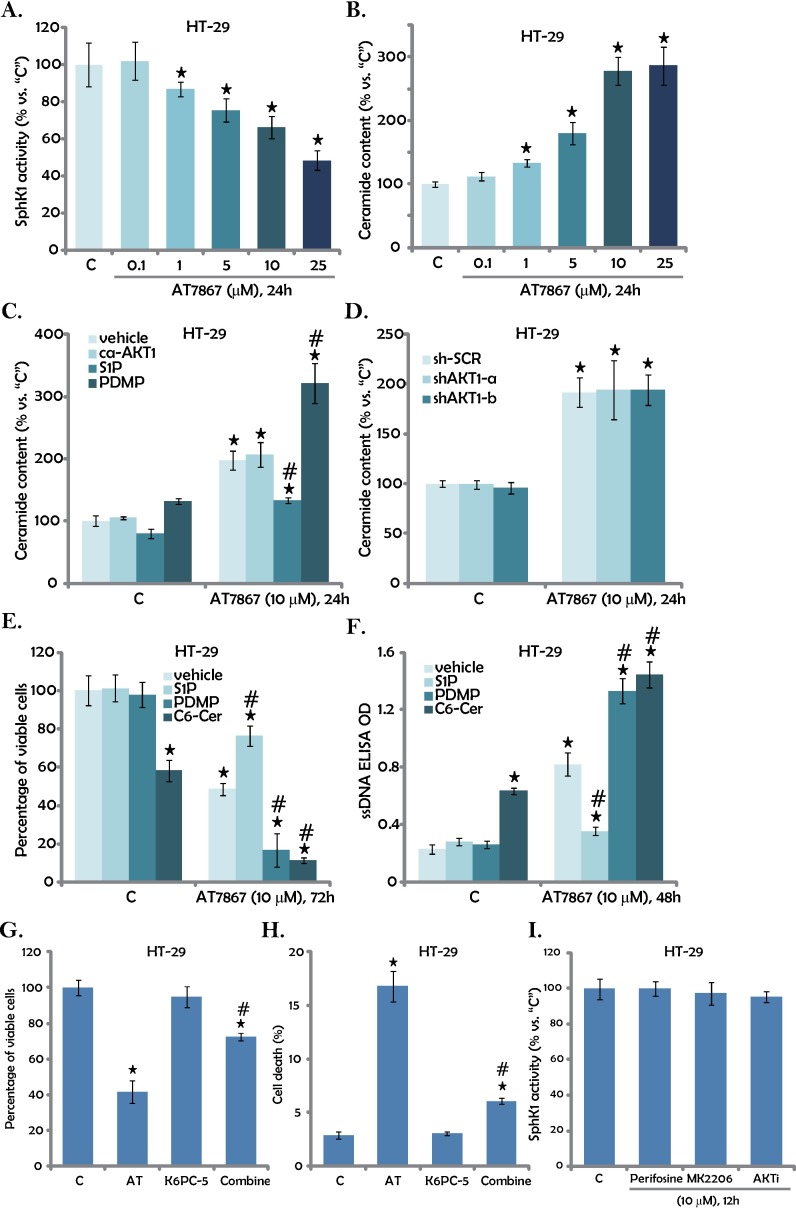
AT7867 inhibits SphK1 to promote ceramide production in CRC cells. HT-29 cells were treated with AT7867 (0.1–25 μM) for applied time; Relative SphK1 activity (A) and ceramide production (B) were tested. HT-29 cells, expressing the ca-AKT1 construct or empty vector, were pre-treated for 1 hour with sphingosine-1-phosphate (“S1P”, 10 μM), L-threo-1-phenyl-2-decanoylamino-3-morpholino-1-propanol (“PDMP”, 25 μM) or C6 ceramide (“C6-Cer”, 25 μM), followed by AT7867 (10 μM) treatment for applied time; Cellular ceramide content (C), viable cell percentage (E, MTT assay) and cell apoptosis (F, ssDNA ELISA assay) were examined. HT-29 cells, expressing AKT1 shRNA (“shAKT1-a/-b”) or scramble control shRNA (“sh-SCR”), were treated with AT7867 (10 μM) for 24 hours, relative ceramide content (*vs*. “C”) was analyzed (D). HT-29 cells were treated with of AT7867 (“AT”, 10 μM) and/or K6PC-5 (10 μM) for 72 hours; MTT assay was performed to test viable cell percentage (G); Cell death was tested by trypan blue staining assay (H). HT-29 cells were treated with 10 μM of perifosine, MK2206 or AKT inhibitor II (“AKTi”) for 24 hours, relative SphK1 activity was tested (I). Data were shown as the mean (n = 5, within the same experiment) with SD. * P < 0.05 *vs*. “C” (medium control) group. ^#^ P < 0.05 *vs*. AT7867 only group.

Above results suggest that AKT-independent mechanisms could also contribute to AT7867-induced cytotoxicity in CRC cells. Growing evidences have indicated that SphK1 is an important oncotarget of CRC [28,29]. Over-expression and/or sustained-activation of SphK1 contributes to cancer cell progression and apoptosis-resistance [30,31]. Inhibition, mutation or silence of SphK1 would increase cellular ceramide production to promote cell apoptosis[30,31]. Interestingly, a very recent study has shown that AKT inhibitor A-674563 could also inhibit SphK1 activity, independent of AKT inhibition [32]. Thus, SphK1 activity and ceramide production in AT7867-treated CRC cells were tested using the methods described previously[17]. Results in Fig 4A showed clearly that AT7867 dose-dependently decreased SphK1 activity in HT-29 cells. SphK1 protein expression was not altered following the AT7867 treatment (Data not shown). Consequently, the cellular ceramide level was significant increased(Fig 4B). Intriguingly, restoring AKT activation by expression of ca-AKT1 (See Fig 3) failed to affectAT7867-induced ceramide production(Fig 4C). Further, a comparable ceramide production by AT7867was noticed in AKT1-silenced HT-29 cells (Fig 4D). These results imply that AT7867-induced ceramide production apparently is independent of AKT inhibition.

To study the role of ceramide production in AT7867-induced HT-29 cell death, pharmacological strategy was applied. Sphingosine 1-phosphate (S1P), an anti-ceramide sphingosine[33], inhibited AT7867-induced ceramide production (Fig 4C), and subsequent HT-29 cell death (Fig 4E) and apoptosis (Fig 4F).Reversely, PDMP, the glucosylceramide synthase inhibitor[34,35,36], facilitated AT7867-induced ceramide production (Fig 4C) and HT-29 cell death and apoptosis (Fig 4E and 4F). These results indicated that ceramide production should also participate in AT7867-induced killing of HT-29 cells. To support this notion, we showed that exogenously-added C6 ceramide (“C6-Cer”) also induced HT-29 cell death and apoptosis (Fig 4E and 4F). More importantly, C6 ceramide further sensitized AT7867-inducedcy to toxicity (Fig 4E and 4F).Based on the results above, we would speculate that the SphK1 activator may attenuate AT7867’s cytotoxicity against CRC cells. Indeed, we showed that K6PC-5, a SphK1 activator [16,37,38], attenuated AT7867-induced HT-29 cell death (Fig 4G and 4H). On the other hand, other AKT inhibitors, perifosine, MK2206 and AKT inhibitor II, failed to inhibit SphK1 activity in HT-29 cells (Fig 4I). These results further suggest that SphK1 inhibition and ceramide production, independent of AKT inhibition, should also contribute to AT7867-induced cytotoxicity against CRC cells.

### 3.5. AT7867 inhibits HT-29 tumor growth in nude mice

**Fig 5:**
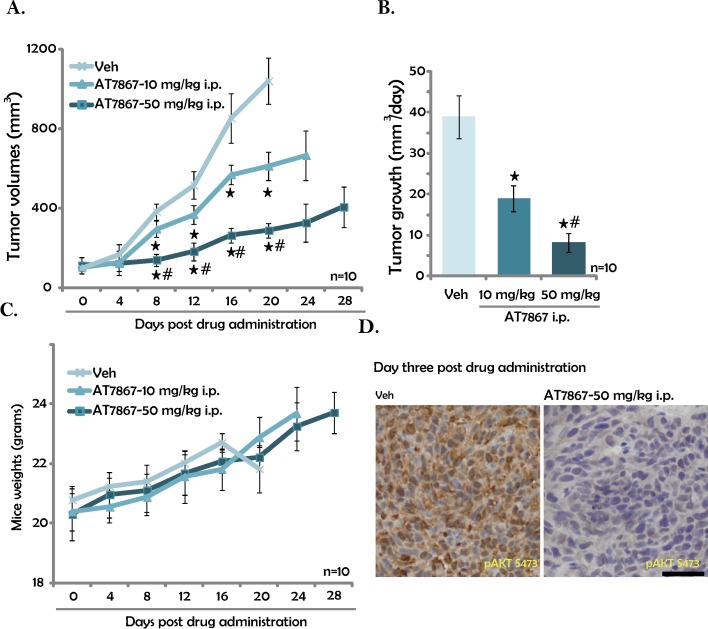
AT7867 inhibits HT-29 tumor growth in nude mice. HT-29 tumor-bearing nude mice (n = 10 per group) were administrated with AT7867 (10 or 50 mg/kg body weight, *i*.*p*., daily for 16 days) or vehicle control (“Veh”); Tumor volumes (A) and mice body weights (C) were recorded ever four days; Estimated daily tumor growth (mm^3^/day) was also shown (B). Three days after initial drug administration, pAKT (Ser-473) in “Veh” and AT7867 (50 mg/kg)-treated HT-29 tumors was tested by the IHC staining assay, representative images were presented (D). Data were shown as the mean with SD.* P < 0.05 *vs*. “Veh” group. ^#^ P < 0.05 *vs*. AT7867 only group. Bar = 75 μm (D).

In order to examine the effect of AT867 on tumour growth *in vivo*, HT-29 cells were injected *s*.*c*. to the nude mice to establish the xenograft tumors. Tumor growth curve results in Fig 5A displayed that *i*.*p*. injection of AT7867 (10 and 50 mg/kg) significantly inhibited HT-29 tumor growth in nude mice. AT7867 at 50 mg/kg was more efficient than 10 mg/kg in inhibiting HT-29 tumor growth (Fig 5A). Estimated daily tumor growth results in Fig 5B further confirmed the significant anti-HT-29 tumor activity byAT7867. Notably, the mice body weight was not significantly affected by the AT7867 administration. Neither did we notice any signs of apparent toxicities. These results, consistent with reports from other studies[15], suggested that the AT7867 treatment regimens here were relatively safe to the mice. IHC staining assay results in Fig 5D demonstrated that pAKT level was significantly lower in the AT7867 (50 mg/kg)-treated HT-29 tumors. Together, these results show that *i*.*p*. injection ofAT7867efficientlyinhibits HT-29 tumor growth in nude mice.

## 4. Discussion and Conclusions

Several mechanisms are responsible for the sustained activation of AKT in CRC and other malignancies, including constitutive activation of upstream receptor tyrosine kinases (*i*.*e*. EGFR), *PIK3CA/PTEN* mutations, *AKT* amplification and/or mutation[7,8]. These will lead to persistent growth of CRC cells[7,8]. Thus, pharmacological inhibition of AKT represents a rational approach for the treatment of CRC [7,8]. A number of AKT inhibitors of different mechanisms of action have recently been developed[7,8].In the current study, we showed that AT7867, a novel AKT and S6K1 dual inhibitor[15], inhibited survival and proliferation of established and primary human CRC cells. Meanwhile, AT7867 provoked caspase-dependent apoptosis in CRC cells. *In vivo*, AT7867 *i*.*p*. injection suppressed HT-29 xenograft tumor growth in nude mice.

AT7867 blocked AKT-S6K1 signaling in CRC cells. However our results indicated that AKT-S6K1inhibition is unlikely the sole mechanism responsible for AT7867-mediated cytotoxicity in CRC cells. First, exogenous expression of ca-AKT1 completely restored AKT-S6K1 activation in AT7867-treated HT-29 cells, but only partially inhibited HT-29 cell death. Second, in AKT1-silenced HT-29 cells, AT7867 was also cytotoxic and pro-apoptotic.

In line with previous studies [15], we showed that AT7867 inhibited AKT and S6K1 activations in both established and primary human CRC cells. Besides, we here proposed a novel mechanism to explain its cytotoxicity against CRC cells. AT7867 inhibited SphK1 to promote pro-apoptotic ceramide production in CRC cells. This AKT-independent mechanism also participated in AT7867-induced anti-CRC cell activity.S1P, which inhibited AT7867-provoked ceramide production, also attenuated subsequent HT-29 cell death. Reversely, PDMPor C6 ceramide significantly augmented AT7867-induced HT-29 cell death.K6PC-5, a SphK1 activator, attenuated AT7867-induced HT-29 cell death (Fig 4). This should also explain why AT7867 was more efficient than other specific AKT inhibitors (perifosine, MK2206 and AKT inhibitor II) in killing CRC cells. As these other AKT inhibitors failed to affect SphK1 activity (Fig 4). It could also be the reason of in-effective of this compound in normal colon epithelial cells. Indeed, AKT and S6K1 expression/phosphorylation as well as SphK1 expression were significantly lower in these epithelial cells, as compared to HT-29 CRC cells (S1 Fig).

Intriguingly, AT7867-induced ceramide production appears independent of AKT inhibition. As ca-AKT1 failed to inhibit AT7867-indued ceramide production. Meanwhile, in AKT1-silenced HT-29 cells, a compare ceramide production was still noticed. In summary, the results of this preclinical study suggest that AT7867 inhibits human CRC cells *in vitro* and *in vivo*. Both AKT-dependent and AKT-independent (*i*.*e*.SphK1 inhibition and ceramide production) mechanisms could be responsible for its superior actions against CRC cells.

## Supporting Information

S1 Fig Expressions of listed proteins in both HT-29 cells and the primary human colon epithelia cells (“Epi”)were shown. Relative expression of the proteins (vs. Tubulin) was quantified.
(EPS)
